# Investigating Process–Structure–Property Relationships in Large-Scale Additively Manufactured Carbon-Filled PETg

**DOI:** 10.3390/ma19112270

**Published:** 2026-05-27

**Authors:** Christopher Bock, Brett Ellis, Masoud Rais-Rohani

**Affiliations:** 1 Advanced Structures and Composites Center, University of Maine, 35 Flagstaff Road, Orono, ME 04469, USA; christopher.c.bock@maine.edu; 2 Department of Mechanical Engineering, University of Maine, 75 Long Road, Orono, ME 04469, USA; 3 Department of Mechanical Engineering Technology, University of Maine, 5711 Boardman Hall, Orono, ME 04469, USA; brett.ellis@maine.edu

**Keywords:** additive manufacturing, thermoplastic composites, extrusion-based additive manufacturing, microstructure, mechanical properties

## Abstract

Properties of a material fabricated by large-scale additive manufacturing exhibit strong process dependence by way of processing and microstructure. This study seeks to experimentally evaluate this process–structure–property linkage for carbon-fiber-reinforced PETg. To facilitate this investigation, an experimental design involving eight different combinations of layer height, feed rate, bead spacing and screw speed in the printing process is considered. Forty-five microstructure specimens are excised and imaged to reveal the fiber orientation and porosity, and nearly 180 flexural samples are tested to evaluate their strength and stiffness. Measured mean values for modulus along the bead range from 13.3 to 18.6 GPa, and for strength, it is between 158 to 189 MPa. Mean values for the inter-layer stiffness range from 2.9 to 3.2 GPa, and for strength, it ranges between 31.4 and 45.0 MPa. Results indicate a strong relationship between screw speed and fiber orientation and between fiber orientation and stiffness and strength. Additional insights into the fracture behavior of the material are provided using high-speed photography of the moment of fracture and microscopy of the fracture surfaces. This work provides a cohesive process–structure–property dataset that can be used as a reference for validation of process–structure, structure–property, and process–structure–property models.

## 1. Introduction

In large-scale additive manufacturing (LSAM), large quantities of material are extruded in a large build volume (≫1 m^3^), enabling the production of large structures like a house [1], boat [2], or car [3]. Despite LSAM being a promising technology for manufacturing, it is not without challenges. For example, Akbari et al. [4] pointed to multiple cases of delamination of the layers during the printing process. In modeling warpage and residual stresses, Bock et al. [5] found that the process-dependent material properties and process-induced failure modes (e.g., warpage, delamination) needed careful consideration in process and product design. LSAM is suitable for a wide range of polymeric material systems [6], including PETg, Nylon, ABS, ASA, and PLA [7]. Fillers, including wood flour, glass fibers, and carbon fibers may be added to reduce costs, facilitate processing, or improve mechanical performance [8]. Carbon fibers are commonly employed in high-performance applications as their negative or near-zero coefficient of thermal expansion (CTE) in the fiber direction can reduce warpage [9] while simultaneously increasing strength and stiffness. Additionally, Pintos et al. [10] showed that greater fiber lengths increased the stiffness and strength of extruded carbon-fiber-reinforced polymeric materials.

The process–structure–property–performance (PSPP) paradigm [11,12] is a common framework for interpreting and framing causal relationships in design and manufacturing, where the process influences the microstructure, which drives the properties that affect the performance of the as-manufactured part. PSPP relations relevant to additive manufacturing (AM) include the volumetric flow rate [13] and feed rate affecting bead shape and fiber orientation within a bead [14]. Furthermore, the temperature of the extrudate influences the rheology of the material [15], whereas swirl from the screw may influence fiber orientation [16]. Prior studies have explored models for the material flow [17], thermal behavior [18], and micro-mechanics [19]. Fiber alignment affects stiffness, strength, CTE, thermal conductivity, and degree of anisotropy [11,17]. The inter- and intra-bead voids also influence stiffness, strength, and other properties. Clearly, modeling the AM process is a complex problem encompassing rheology, machine design, motion control, chemistry, thermal science, and mechanical behavior of materials.

Additionally, certain polymers can exhibit crystallinity [20,21,22] which exhibits a bi-directional coupling with thermal history [23] and flow [24]. Crystallinity can also have a significant effect on the as-printed properties of the material, where a greater degree of crystallinity typically results in greater stiffness [25]. Crystallinity is negligible in amorphous materials, such as carbon fiber polyethylene terephthalate glycol (cfPETg), and thus may be ignored [26].

Material properties influence part performance in various operating conditions. For example, material strength influences the load-bearing capacity of parts, and CTE influences the dimensional stability [5] and warpage. Both properties influence the occurrence of delamination [3] along with inter-layer strength, where layers split during or after the printing process because of residual thermal stresses and a lack of strength in the build direction. Additionally, fiber content and orientation have an effect on the CTE [27,28]; thus, characterizing the effects of process on microstructure is very important for a priori warpage prediction.

The inter-layer strength is of particular importance. The temperature history at the interface between two adjacent layers influences the geometry of the inter-layer bonds [29,30] and the diffusion [31] of polymer chains across the interface. Allum et al. [32] found that, for the cases considered, the strength of the inter-layer bond were approximately equal to that of the bulk material (for a neat polymer), and the strength was driven by geometry. However, Toth et al. [33] called for further investigation. To aid inter-layer bonding, heating the substrate by laser right before extruding the next layer [34] or annealing parts after printing [20] seem to offer effective strategies, which are attributed to increasing inter-layer diffusion. The effect of process parameters on inter-layer notch geometry and inter-layer strength was evaluated by [35], where the notch geometry and resulting stress concentration were determined to be driving factors in the inter-layer mechanical behavior, the likely reason being a complete inter-layer diffusion. Clearly, the development of inter-layer strength is a highly important goal.

Prior work in this field has indicated inverse correlations between volume extruded and fiber orientation. At small scale, Yan et al. [36] found an inverse relationship between bead width and fiber alignment, which is consistent with the results reported by Consul et al. [37] and Wu et al. [38]. Slattery et al. [39] found an inverse correlation between extrusion ratio and fiber orientation for 10% carbon-filled PLA. At large scale, Talabi et al. [40] found that increasing screw speed generally resulted in a lower degree of fiber alignment, especially away from the bead edges, whereas an increase in shear rate resulted in an increase in fiber alignment. The results for shear rate were consistent with those found by Nguyen et al. [41], who observed that a nozzle with an internal flow constriction resulted in an increased fiber alignment. Simulation work by Seta et al. [42] correlated a lower extrusion speed to higher degrees of fiber alignment, which seemed to be contradictory to the simulation results from Pibulchi et al. [14] where a faster deposition was attributed to a greater degree of alignment through stretching. A recent work by Seta et al. [43] showed that it was possible to exert control over the distribution of fibers within the beads via rotation and inclination of the nozzle relative to the printing surface. The literature suggests multiple fiber orientation causal mechanisms including internal and external flow patterns [33] and fiber–flow interactions.

Talabi et al. [40] also found that, in general, an increase in screw speed resulted in an increase in intra-extrudate porosity, whereas an increase in shear rate was attributed to a decrease in porosity. Work by Tagscherer et al. [44] found that an increase in extrudate temperature resulted in a decrease in porosity. The same trend was found by Sayah and Smith [45] for ABS at large scale, where an increase in temperature was attributed to an increase in shear rate resulting from a decrease in the viscosity of the polymer, which drove the reduction in porosity. Subsequent work by Sayah and Smith [46] found a strong inverse correlation between degree of fiber alignment and porosity. The finding from previous work means the results are in concordance with the finding that higher shear rates result in greater fiber alignment. The results from Sayah and Smith (2022) [45] contrast with results from Tikhani et al. [47] for glass-filled PC at small scale, where an increase in temperature was not attributed to a reduction in intra-bead porosity.

Fiber-reinforced material properties have been modeled via Mori–Tanaka [48], complex variable methods [49], finite element analysis [50], and spectral methods [51]. Fiber diameters are typically an order of magnitude smaller than inter-bead voids. Hence, the two relevant length scales—microscale (fibers and intra-bead voids) and mesoscale (inter-bead voids and interfaces)—motivate the adoption of multiscale methods [29].

Several researchers have investigated the effect of process parameters on properties. Abadi et al. [52] found that extruded material temperature and raster spacing were key factors driving the inter-raster strength of the material. Honarvar et al. [53] investigated the effects of processing on the strength of 30% glass-filled polypropylene and generally found an increase in layer height to result in a decrease in strength, with effects of the screw speed on the longitudinal strength being minimal.

The objective of this work was to experimentally investigate the process–structure–property (PSP) relationships in large-scale AM of cfPETg by exploring the microstructure and mechanical properties (strength and stiffness) for multiple points in process space. The results of this study can serve as a reference supporting the validation and creation of process–structure and structure–property models for design applications. One of the novel aspects of this work is the volume of data points. With nearly 180 flexure tests and 45 microstructure evaluations, the scale and cohesion of the work performed permit multi-point and multi-stage validation of physics-based PSP models, as well as training of regression-based machine learning models. The other novel aspect of the work is the highlighted chains of causality from process to structure to property, and demonstration that the results are a good reflection of the representative microstructure and properties at various points in the process space.

How do process parameters influence the properties of LSAM cfPETg, and in turn, what mechanisms drive the variation in structure and its effect on the overall mechanical properties are the main research questions this work sought to answer. The remainder of the paper is organized as follows: Material processing and characterization through a detailed workflow involving mechanical testing and microscopy are discussed in Section 2. The results obtained from various tests are presented in Section 3, with discussion of results and conclusions following in Section 4 and Section 5 respectively.

## 2. Material Processing and Characterization

**Figure 1:**
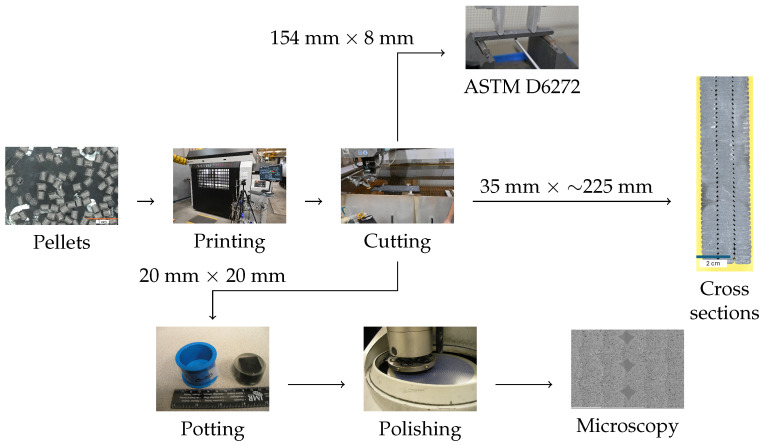
Workflow from process to characterization of properties.

A graphical overview of the workflow from process to experimental characterization of properties is shown in Figure 1.

Techmer (Clinton, TN, USA) Electrafil PETG 1711 3DP [54] (cfPETg 20% milled carbon fibers by volume) resin pellets were dried in a DRI-AIR (East Windsor, CT, USA) dryer at 65 °C for at least 4 h prior to all printing. Parts were printed using a Juggerbot (Youngstown, OH, USA) Tradesman F3-44, a large scale h-bot, having an enclosed 1219 × 914 × 1219 mm^3^ build volume, a 6 mm diameter nozzle, and a Strangpresse (Warren, OH, USA) Model 19 [55] extruder having an ∼8 kg/h maximum extrusion rate. The bed temperature was set to 60 °C for all prints.

Printed parts were rounded rectangles with three-bead-wide wall thicknesses. Because of changes to the process parameters, as-printed dimensions varied from one printed part to another. The exterior envelope of each rounded rectangle was approximately 1000 mm long × 260 mm wide with 30 mm corner radii. The printed parts, of approximately 230 mm in height, comprised an approximately 3 mm tall “foot” in which feed rate was reduced by 50%, a 47 mm tall region to allow the printer to avoid transient effects, a 175 mm tall region for sampling, and at least a 5 mm tall region between the top of the highest specimen and the top of the print. During printing, the upper, middle, and lower extruder heat bands were set to 207 °C, 217 °C, and 228 °C, respectively; the nozzle temperature was set to 238 °C.

Experimental design considered three independent processing parameters—bead spacing $b_{s}$, layer height $d_{h}$, and feed rate *f*—and one dependent processing parameter—screw speed ω—discretized and combined into eight parameter combinations. Initial parameter combinations were determined via a Lloyd-optimized Latin hypercube [56]. Final parameter combinations were determined after qualitative testing and are shown in Table 1. Parts were printed in a random order to minimize confounding resulting from temporal factors (e.g., screw-speed calibration). In Table 1, die swell $d_{s}$ [36] equals the bead spacing (mm) divided by the nozzle diameter (6 mm); aspect ratio $a_{r}$ equals the bead spacing (mm) divided by the layer height (mm). A custom G-code program was generated for each part via a script written in Python 3.11 Die swell, aspect ratio, and feed rate were explicitly considered in the experimental design to ensure printability. The screw speed was calibrated manually before each print and is considered as a dependent variable for the purposes of the experimental design.

The temperature of the substrate layer at the current deposition location is defined as the $T_{2}$ temperature. Ideally, $T_{2}$ is sufficiently cool so the substrate can support the weight of the to-be-deposited extrudate, but sufficiently warm to ensure minimal residual stresses between the layers and sufficient diffusion of polymer chains between layers [30,31,32]. Based upon an analytical model, feed rates were chosen to ensure $T_{2}$ ≥ 80 °C resulting in layer times between 1 and 3 min.

**Figure 2:**
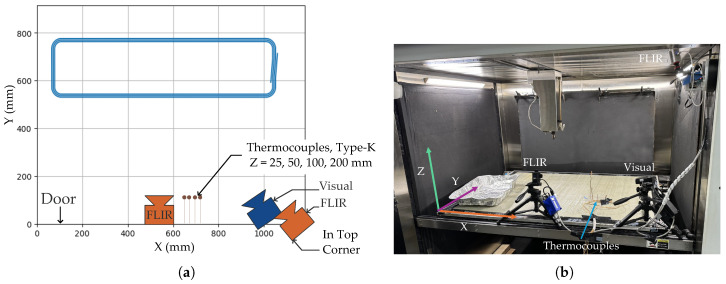
(**a**) Instrumentation setup and (**b**) as-installed layout.

During printing, temperatures were recorded via five K-Type thermocouples and two FLIR Boson thermal cameras. Four of the K-Type thermocouples were located within the printer’s enclosure, and one K-Type thermocouple was located outside the printer enclosure. All thermocouples transmitted data to a Graphtec Model GL240 datalogger. Thermocouple readings were verified by comparing readings to an Omega (Norwalk, CT, USA) HH502 calibrated thermometer. The two FLIR Boson thermography cameras [57] recorded thermographic data of each printed part. Recognizing there are several confounding factors affecting thermography readings including reflected radiation, directionality of the emissivity, and noisy readings, thermography data provided relative measurements of temperature variation. A visible-light camera placed within the printer’s enclosure recorded a time sequence of each print; a second visible-light camera faced the human–machine interface (HMI) to confirm the Juggerbot’s positional setpoints. Figure 2a shows a schematic of the infrared (FLIR) and visual (Visual) cameras, thermocouples, and part position (blue rounded rectangle) within the print chamber. Figure 2b shows the as-installed physical layout.

**Figure 3:**
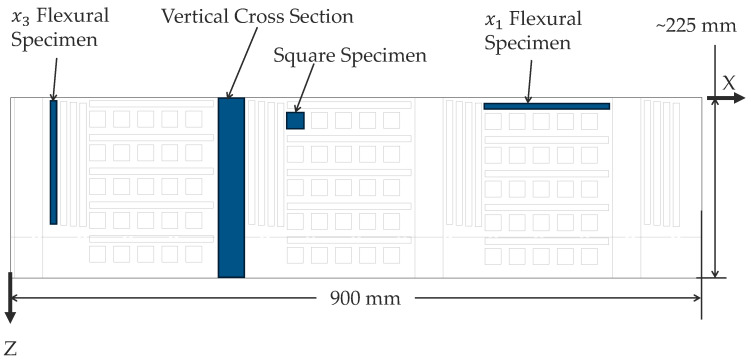
Image showing segment cut plan as well as the cutting coordinate system. Beads are aligned with the X axis.

Each of the eight printed parts were segmented to excise a 900 mm × 230 mm segment. Segments were further processed to obtain 15 $x_{1}$ four-point-bending specimens with their major axis in the bead direction X (153.6 mm × 8 mm × 3 beads), 16 $x_{3}$ four-point-bending specimens with their major axis in the build direction Z (153.6 mm × 8 mm × 3 beads), and 75 square specimens (20 mm × 20 mm × 3 beads). Three of the square specimens were used for density testing. At 3 beads wide, specimen widths ranged from approximately 18 mm to 26 mm (cf. Table 1). A subset of manufactured specimens were tested. An image of the segment cut plan is shown in Figure 3, noting the cutting coordinate system is different from the printer coordinate system.

### 2.1. Microstructure Analysis

#### Microscopy

The polished microscopy specimens were imaged on a Keyence (Osaka, Japan) VHX-7000H optical microscope featuring auto-focus, using full coaxial lighting at a 150× magnification, resulting in a resolution of 2880 × 2160 pixels per image. Hence, the microscope captured approximately 15 pixels across a fiber’s 5 μm diameter.

**Figure 4:**
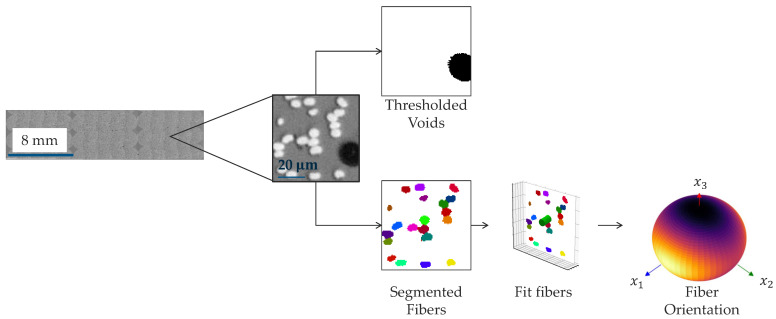
Microstructure extraction procedure.

A graphical overview of the process to extract data from microscopy images is shown in Figure 4.

Fiber orientation was evaluated by identifying pixels in a fiber by thresholding and using a watershed step to distinguish individual fibers, the colors shown have no significance. Orientations were fit to the fibers as described in Advani and Tucker [58] and Sharp et al. [59] for elliptical fibers. Normally, a non-uniform weighting function is used to account for fibers normal to the imaging plane having a higher probability of being captured [19], hence the use of the Konieck weighting function. Microstructure measurements were taken over the entirety of the 20 mm × 3 bead cross section.

**Figure 5:**
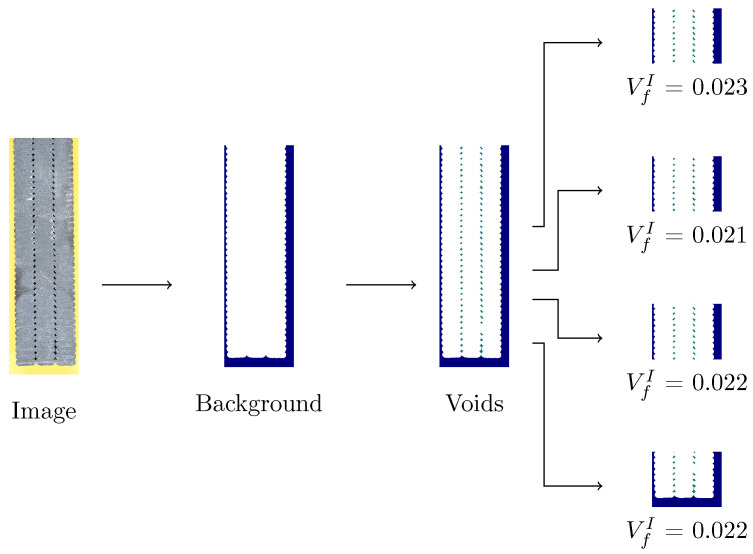
Macro image analysis (Print 4).

Intra-bead porosity was calculated by thresholding fibers via a Gaussian blur [60]. This approach produced a binary image, which determined what pixels were within a bead B(ι). To find voids, the original image was subjected to a threshold again with 5 additional erosion and dilation steps to eliminate artifacts caused by shadows occasionally found around fibers. This procedure produced a binary image identifying whether certain pixels were within an intra-bead void V(ι). The volume fraction $V_{f}^{i}$ of intra-bead voids was found using

(1) $$V_{f}^{i}=\frac{\sum_{\iota \in I}\left(B\left(\iota \right)\wedge V\left(\iota \right)\right)}{\sum_{\iota \in I}B(\iota )},$$

where ∧ is the logical and operator, and *I* is the set of pixels. The numerator of Equation (1) determines the number of pixels inside a bead and in a void, whereas the denominator is the number of pixels in a bead. The vertical cross-section specimens were prepared and imaged using a Nikon (Tokyo, Japan) Z30 20 MP camera with a 50 mm f/2.8 macro lens and a ring flash. Nominally, the resolution was approximately 30 pixels/mm. Macro images were analyzed to evaluate inter-bead porosity via the workflow shown in Figure 5. As shown in Figure 5, inter-bead void fractions were quantified by thresholding cross sections, determining background, identifying voids via pixel luminance, and removing void regions having fewer than 50 connected pixels representing intra-bead voids. Lastly, the remaining void regions were de-noised. The inter-bead porosity can be found by taking the ratio of void pixels to the total number of non-background pixels in the cross section. This was done for four evenly sized horizontal slices per image.

### 2.2. Four-Point Bending

**Figure 6:**
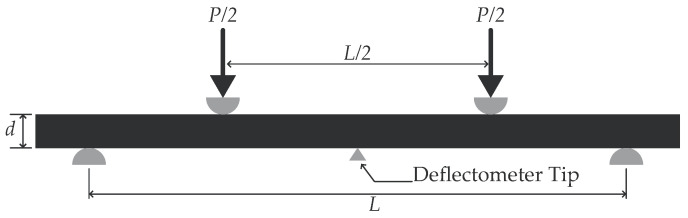
Specimen geometry and boundary conditions, *d* = 8 mm, *L* = 128 mm.

Four-point bending tests of $x_{1}$ and $x_{3}$ specimens were conducted in accordance with ASTM D6272 [61] and ASTM D618 [62] Procedure A, with the nominal specimen geometry and loading locations shown in Figure 6. The thickness of each specimen was measured thrice at five locations using a Mitutoyo micrometer with flat anvils; the width of each specimen was measured thrice at three locations using a Starrett micrometer with an alignment jig and flat anvils. Calculations used the average thickness and width measurements of each specimen.

**Figure 7:**
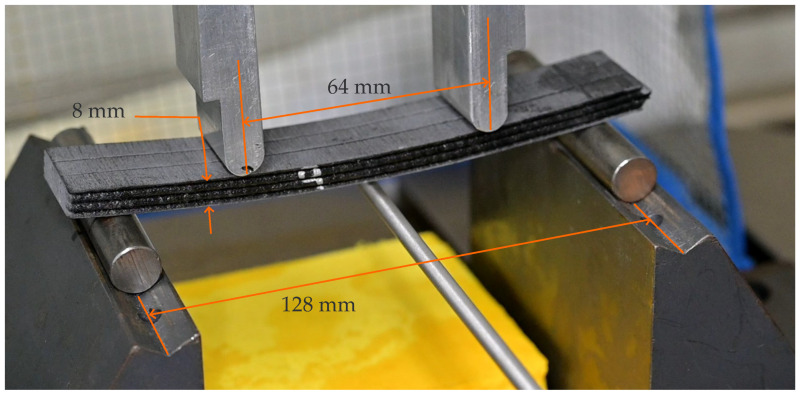
Testing setup for four-point bending experiments.

Specimens were tested on an Instron (Norwood, MA, USA) hydraulic frame at a 3.4 mm/min loading rate. An Epsilon Technologies (Jackson, WY, USA) 3540-025M-ST deflectometer, having a 25 mm maximum displacement, measured each specimen’s vertical deflection at midspan. An image of the testing setup is shown in Figure 7.

A Photron (Tokyo, Japan) SA4 high-speed camera, captured the failure response of representative 4-point-bending specimens at 40,000 frames per second at approximately 0.05 MP. As a result of the extremely low exposure in the raw images, gamma correction was performed using scikit-image [60]. The maximum bending stress at a beam’s midspan, and the flexural moduli were determined in accordance with ASTM D6272 [61].

### 2.3. Density Measurements

Three 20 mm × 20 mm × 3 bead square specimens were excised from each print for density measurements. The mass of each specimen was measured via an O’Haus Explorer EX224N analytical balance having a 0.001 g resolution. The volume of each specimen was measured via an Anton Paar (Graz, Austria) Ultrapyc 5000 Foam gas pycnometer in a 23 °C helium atmosphere at 131 kPa pressure. Pycnometer measurements ceased after a variance of ≤0.05% was reached. Density ρ was calculated as the mass divided by the volume.

## 3. Results

**Figure 8:**
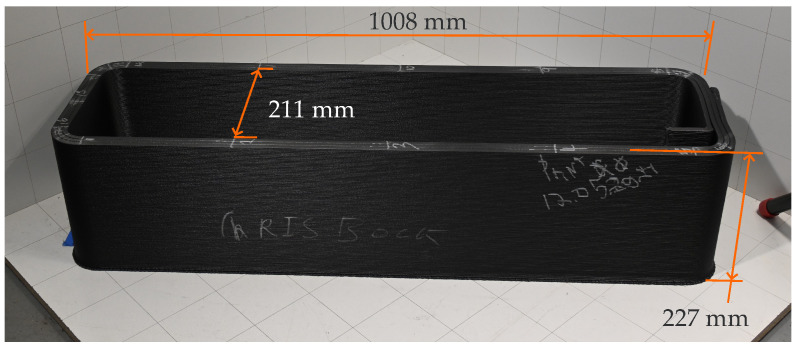
Print 1 (gridlines at 10 cm).

A total of eight parts were printed (cf. Figure 8). In total, 177 four-point bending specimens, 44 microscopy, and 24 density specimens were utilized.

**Figure 9:**
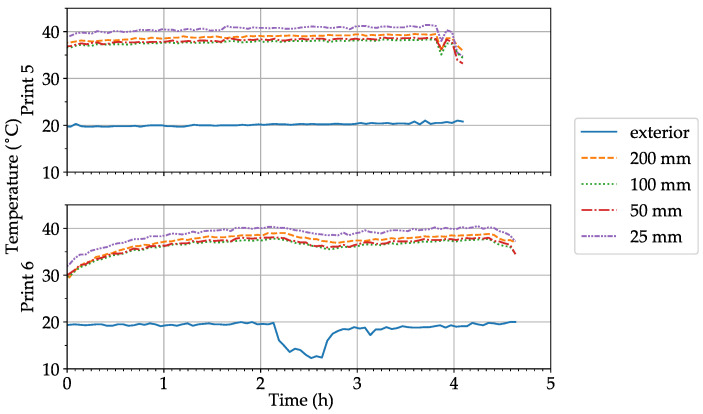
Interior and exterior ambient temperature variations for Prints 5 and 6.

Interior and exterior temperature profiles for Prints 5 and 6 are shown in Figure 9 as examples. The interior temperatures measured at 25 mm were approximately 5 °C greater than the interior temperatures measured at 200 mm. The temperature profiles for Prints 1–4 and Prints 7–8 were similar to that of Print 5; however, the transient fluctuation in temperature during Print 6 from 2 h:10 m to 2 h:45 m was due to a reduction in the temperature immediately outside the printer. This observation indicates that even in an ostensibly closed print volume, a change in exterior temperature can influence the temperature of the interior print volume.

With the experimental limitations in mind, the relationship between $T_{ambient}$ (interior of the printer) and layer time *t* on $T_{2}$ can be approximated using a lumped parameter heat transfer model considering a single bead subject to convection on the top, left, and right sides [63]:

(2) $$T_{2}\approx \left(T_{n}-T_{ambient}\right)\exp\left(-\frac{(2d_{h}+b_{s})ht}{\rho c_{p}b_{s}d_{h}}\right)+T_{ambient}$$

where $T_{n}$ is the nozzle temperature, *h* is the convection coefficient, ρ is the density, $c_{p}$ is the specific heat, and $T_{ambient}$, $d_{h}$, and $b_{s}$ were previously defined.

For a specific combination of parameter values (i.e., $\rho =1328\;\mathrm{kg}/m^{3}$, $b_{s}=7.742$ mm, $d_{h}=1.64$ mm, $c_{p}=1120\;W/\mathrm{kg}\;{}^{\circ}C$, $h=8\;W/m^{2}\;{}^{\circ}C$, $T_{ambient}=40\;{}^{\circ}C$, and layer time t=100 s, nozzle temperature $T_{n}=238\;{}^{\circ}C$), changing $T_{ambient}$ by +/− 10% would cause $T_{2}$ to change by approximately 3 °C, whereas altering the layer time by +/− 10% would change $T_{2}$ by 11.5 °C. Because the lumped parameter model neglects conduction between adjacent beads and layers, the effect of $T_{ambient}$ is further reduced. Thus, the minor fluctuation in $T_{ambient}$ observed in Print 6 appears to have a negligible effect on other factors that might contribute to inter-layer diffusion and inter-print differences resulting from the process-parameter variation.

**Figure 10:**
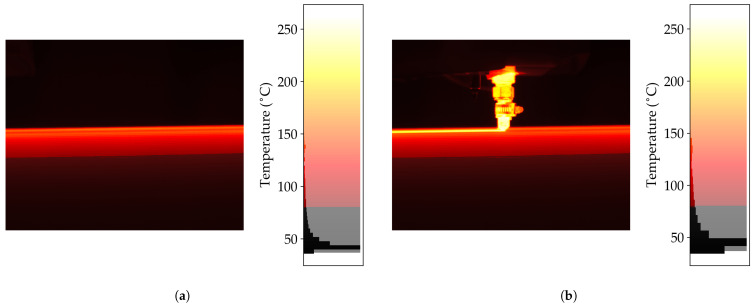
FLIR infrared radiation images of Print 3 at *t* ≈ 3.1 h (**a**) before deposition and (**b**) during deposition of subsequent layer.

The infrared radiation images and the associated temperature histograms for Print 3 at two instants in time near *t* ≈ 3.1 h are shown in Figure 10. In Figure 10a, the FLIR-estimated temperature of the substrate cfPETg bead is approximately 100 °C, which is well above the 75 °C glass transition temperature of the polymer [64], meaning good inter-layer adhesion is expected [20]. It should be noted that inter-layer adhesion is not only driven by $T_{2}$ but also by the time above $T_{g}$, interface pressure, surface chemistry, and fiber position, as they all matter when considering the strength of inter-layer interfaces. The same was true for the other prints, where $T_{2}$ was far above $T_{g}$.

### 3.1. Microstructure

**Figure 11:**
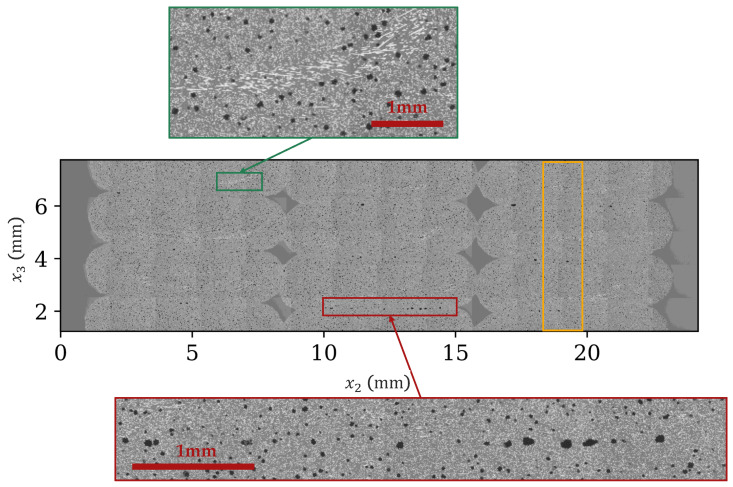
Sample microstructure of Print 1.

A stitched composition of Print 1 from multiple microscopy images at X = 216 mm and Z = 82 mm is shown in Figure 11. The vertical dark-colored ridges, one boxed in orange, are a result of slightly different lighting conditions for the stitched images and not actual ridges in the microstructure.

Figure 11 also captures the layer interface voids as highlighted in the red box. Compared to the inter-bead voids, the interface voids lack potting resin, indicating the interface voids are non-surface-connected. Both interface and inter-bead voids are expected to reduce the inter-layer strength of the print. Another visible feature is the region of fiber misalignment (cf. green box) located in roughly the same location in each bead, which indicates some flow condition at this location is causing the fibers to align in the $x_{2}$ direction. This is likely due to the screw rotation, causing one side of the bead to have slightly different flow conditions, hence the asymmetry.

**Figure 12:**
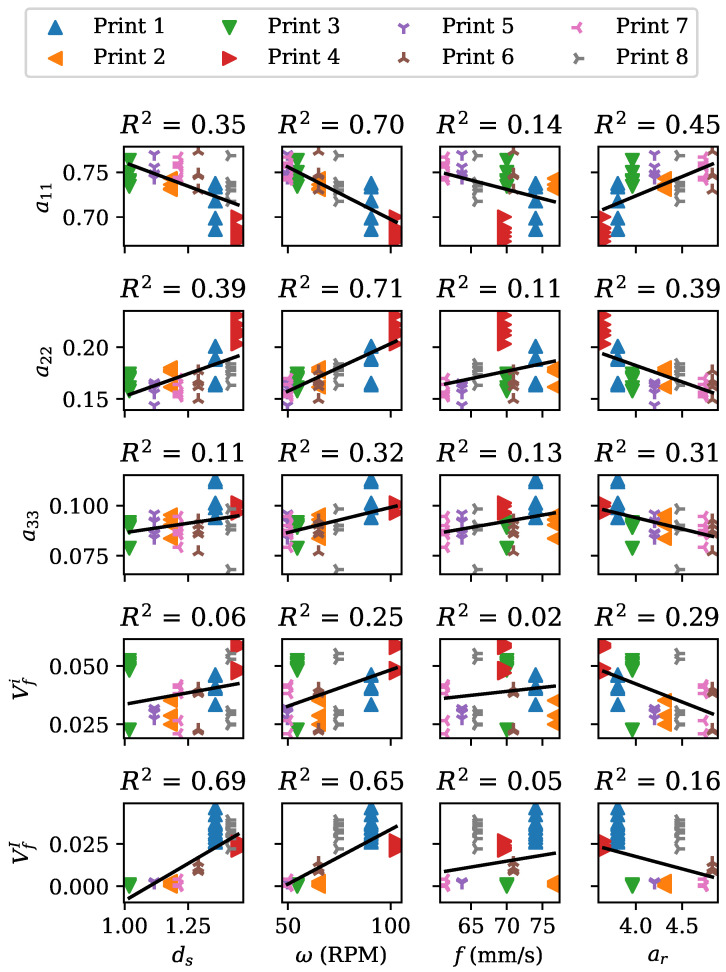
Effect of process parameters on structure parameters.

The plots in Figure 12 illustrate the effect of the individual process parameters on the microstructure through orientation tensor components $a_{11},a_{22},a_{33}$, inter-bead porosity $V_{f}^{I}$, and intra-bead porosity $V_{f}^{i}$. The calculated coefficient of determination, $R^{2}$ for a linear correlation estimate ranges from a low of 0.02 to a high of 0.71, suggesting a lack of linear relationship in most plots.

The lower bound of zero for inter-bead void $V_{f}^{I}$ is indicative of samples having either no such voids or voids being too small to resolve with a camera. Increasing bead spacing $d_{s}$ appears to increase $V_{f}^{I}$ and decrease the degree of fiber alignment $a_{11}$. Increasing screw speed ω appears to be detrimental to both inter-bead void and fiber alignment. Feed rate *f* seems to be influential on fiber alignment but not so much on the remaining microstructure parameters.

Microstructure results, save for $V_{f}^{I}$, are tabulated in Table 2. The overall average values are shown in the bottom row. Among the microstructure parameters, intra-bead void $V_{f}^{i}$ shows the largest variability, which is in contrast to fiber alignment $a_{11}$. The high coefficient of variation (COV) values in $V_{f}^{i}$ is attributed to random fluctuations in the porosity.

Inter-bead porosity measurements are summarized in Table 3. The variance for some of the prints is high because the inter-bead porosity is so small in some prints, with noise in the measurement being of a similar magnitude to the actual measured value.

**Figure 13:**
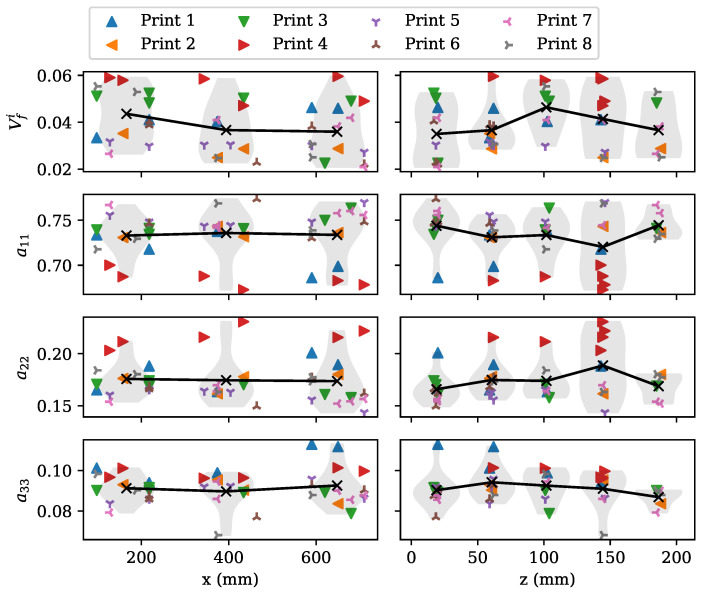
Effect of cut position on structure.

Figure 13 shows the effect of sample location (with associated coordinates) on the measured microstructure parameters. The solid line in each plot highlights the average values within each cluster. The light gray shape is a violin plot of the properties at a particular location cluster. Considering the range of values for each microstructure parameter, the effect of location appears to be fairly modest, indicating the process is operating in a quasi-steady-state regime, where the process is behaving “nominally”.

### 3.2. Four-Point Bending

**Figure 14:**
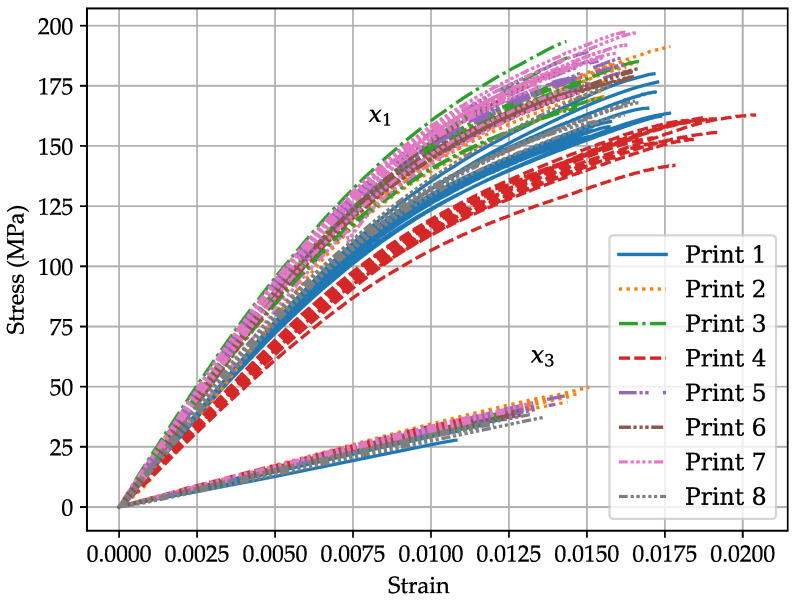
Flexural stress–strain curves from four-point bending tests.

In total, 80 (D6272) specimens in the $x_{1}$ direction and 97 specimens in the $x_{3}$ direction were tested. The engineering stress–strain curves for the flexural specimens are shown in Figure 14. The flexural response in the $x_{1}$ direction appears to transition from linear to nonlinear at a strain of approximately 0.005, whereas the flexural response in the $x_{3}$ direction remains linear up until failure.

The mean and coefficient of variation values of flexural moduli and ultimate flexure strengths for $x_{1}$ and $x_{3}$ samples from eight separate prints are shown in Table 4. The last row shows the mean and variability for all the $x_{1}$ and $x_{3}$ samples in two groups of 96 and 80, respectively. Within each print, variability in each property is less than that of the group, as expected.

It is worth noting that whereas the overall mean in $E_{11}$ is over five times greater than that in $E_{33}$, its variability is only twice as much. In contrast, while the overall mean in **$S_{u_{11}}$** is roughly 12% greater than that in **$S_{u_{33}}$**, level of variability in **$S_{u_{11}}$** is about 60% that in **$S_{u_{33}}$**.

The strain at failure for the samples from each print is shown in Table 5. The last row shows the mean and coefficient of variation values for the entire group of samples in $x_{1}$ and $x_{3}$, respectively. The range of COVs for the $x_{3}$ samples appears to be significantly higher than that observed in the $x_{1}$ samples. This difference is attributed to the impact of $V_{f}^{I}$ and $V_{f}^{i}$ on properties in the $x_{3}$ direction.

**Figure 15:**
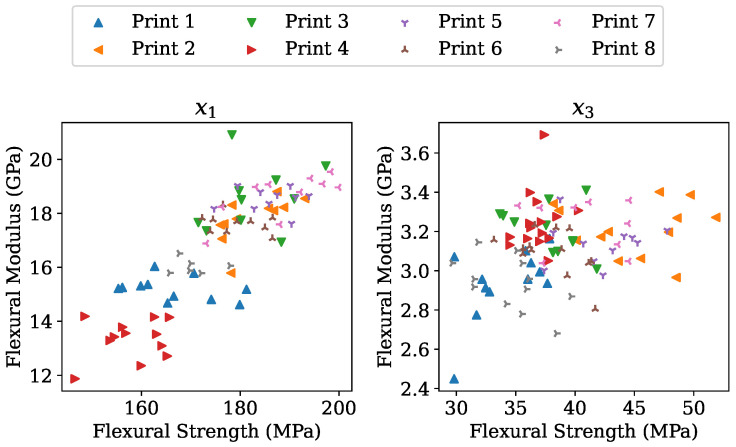
Paired plots of flexural strength and flexural modulus in $x_{1}$ and $x_{3}$ directions.

A paired plot of the flexural strength and flexural modulus for the samples in the $x_{1}$ and $x_{3}$ directions is shown in Figure 15. Whereas, there is a positive correlation between flexural modulus and flexural strength in the $x_{1}$ direction, there does not appear to be any correlation between the same properties in the $x_{3}$ direction. The strong correlation between flexural strength and flexural modulus in the $x_{1}$ direction hints at the failure mode being related to fibers and their general alignment, as expected.

**Figure 16:**
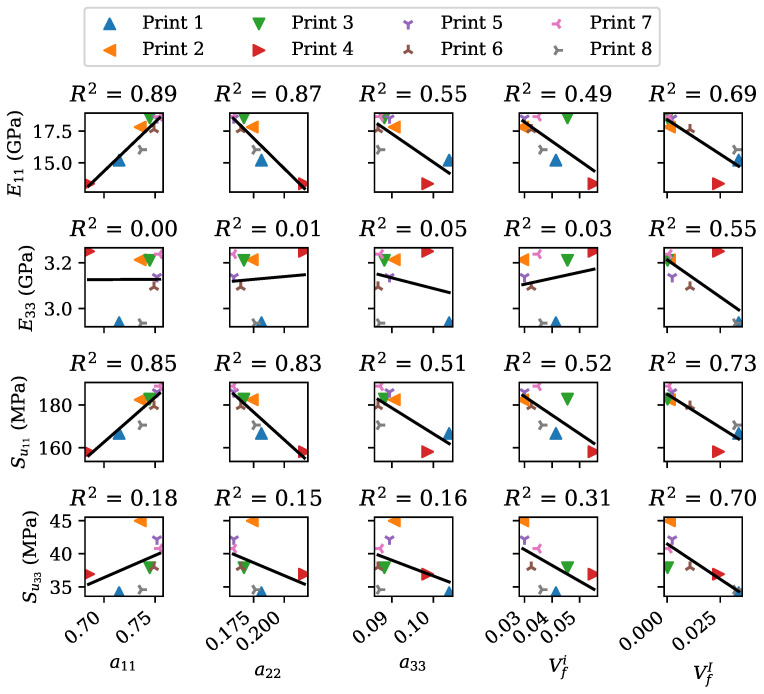
Effect of structure on stiffness and strength.

Figure 16 shows the mean properties ($S_{u_{11}}$, $S_{u_{33}}$, $E_{11}$, and $E_{33}$) as a function of the mean microstructure parameters ($a_{11},a_{22},a_{33}$, $V_{f}^{I}$ and $V_{f}^{i}$) across each print.

The linear correlation approximations and the associated $R^{2}$ values are also shown. There appears to be a strong positive correlation between the orientation tensor component $a_{11}$ and the modulus $E_{11}$ and strength $S_{u_{11}}$. A similarly strong but negative correlation also exists between the other two orientation tensor components ($a_{22}$ and $a_{33}$) and the modulus $E_{11}$ and strength $S_{u_{11}}$. Both inter-bead and intra-bead voids also display a negative correlation with the modulus $E_{11}$ and strength $S_{u_{11}}$, which is also not surprising. As for the modulus $E_{33}$, the microstructure parameter with the highest correlation is $V_{f}^{I}$. Generally, all four material properties are strongly influenced by $V_{f}^{I}$ [32]. The strong correlation between $V_{f}^{I}$ and $S_{u_{33}}$ means a probable mechanism behind the inter-print variation in the properties is the geometry at the inter-layer interface, not a lack of diffusion at the interfaces. The relationship between $V_{f}^{I}$ and $S_{u_{11}}$ is likely a result of the correlation between $V_{f}^{I}$ and $a_{11}$. In this specific case, $a_{11}$ and $V_{f}^{I}$ are correlated with each other as a result of the specific combination of factors, and $V_{f}^{I}$ can be reduced via over-extrusion.

**Figure 17:**
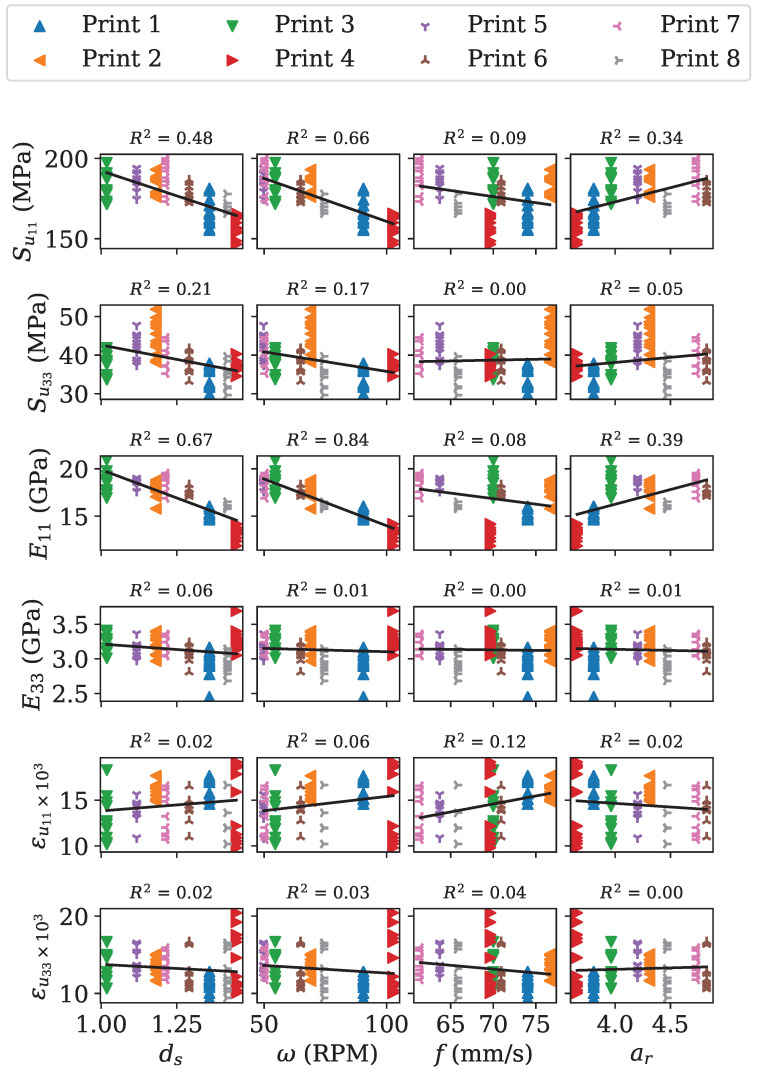
Effect of process on properties.

Figure 17 shows $S_{u_{11}}$, $S_{u_{33}}$, $E_{11}$, $E_{33}$, $\epsilon_{u_{33}}$, $\epsilon_{u_{33}}$ as a function of die swell $d_{s}$, screw speed ω, feed rate *f*, and aspect ratio $a_{r}$. Among the effects examined, the strongest relationship is between ω and $E_{11}$.

**Figure 18:**
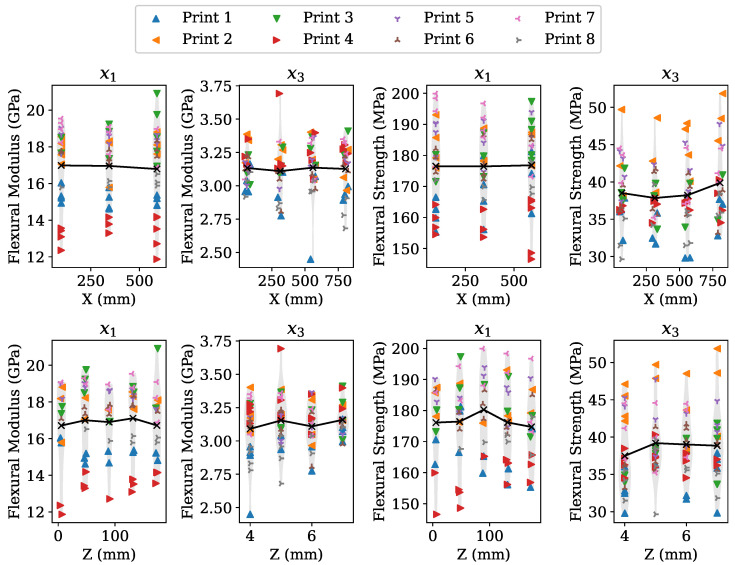
Flexural moduli and strengths as functions of spatial locations in Prints 1 through 8.

X–Z spatial provenance of each sample in the cutting coordinate system (cf. Figure 3) was tracked to determine whether spatial position had any effect on the as-printed properties of the parts. The flexural moduli and flexural strengths as functions of spatial position in the X and Z directions are shown in Figure 18 with the horizontal axis in the top row showing the X-position of the left-most edge of the $x_{1}$ specimens as measured from the top-left corner of each segment; the horizontal axis in the bottom row shows the Z-position of the top-most edge of the $x_{3}$ specimens as measured from the top-left corner of each segment (cf. Figure 3). The solid black line in each sub-figure in Figure 18 passes through the mean values at the specified locations. The light gray shape is a violin plot of the properties at a particular location cluster. While there appears to be noticeable differences in the measured values from one print to another, the combined mean values of the measured properties show little to no significant dependence on the spatial location.

### 3.3. Fracture Behavior of Bending Specimens

**Figure 19:**
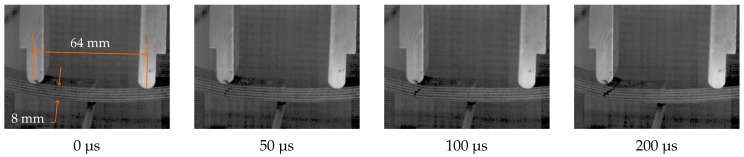
Time lapse images showing fracture of Print 4—$x_{1}$ four-point bending Specimen 9.

The time lapsed high-speed camera footage, as highlighted in Figure 19, shows that the vast majority of the $x_{1}$ bending specimens experienced brittle failure. Additionally, fracture started at the bottom surface of the specimen and propagated upward in stages.

**Figure 20:**
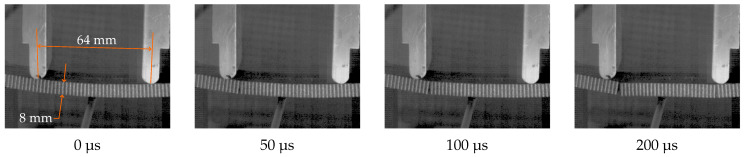
Time lapse images showing fracture of Print 1—$x_{3}$ four-point bending Specimen 9.

The $x_{3}$ failure highlighted in Figure 20, in which the specimen broke in two distinct places along the bond surface between two adjacent layers, is representative of the $x_{3}$ bending specimens. In Figure 20, the first fracture appears near the left loading point at 50 µs. The second fracture (near the right loading point) propagates through the specimen at 200 µs. The failure mode for $x_{3}$ specimens was far more explosive compared to $x_{1}$ specimens.

### 3.4. Fracture Surface of Four-Point Bending Specimens

**Figure 21:**
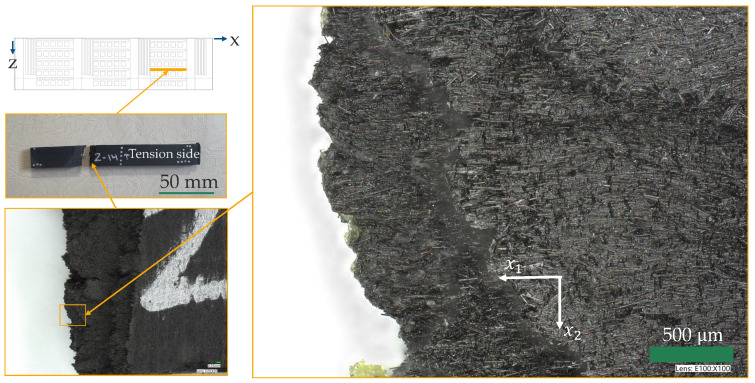
Fracture surface of Print 3—$x_{1}$ four-point bending Specimen 14.

A representative fracture surface for an $x_{1}$ four-point bending specimen is shown in Figure 21. Inspection of the fracture surface in Figure 21 indicates a high degree of fiber alignment, suggesting a high degree of anisotropy. Visible fiber lengths are on the order of 100 µm. The presence of isolated fibers at the fracture surface is an indication that fiber pullout was a factor in the failure of the specimen. Although mating holes on the adjacent fracture surface were not resolvable, the fibers and their alignment were driving factors behind the strength in the $x_{1}$ direction. Print IDs were internally indexed at zero, hence the marking on the specimen.

**Figure 22:**
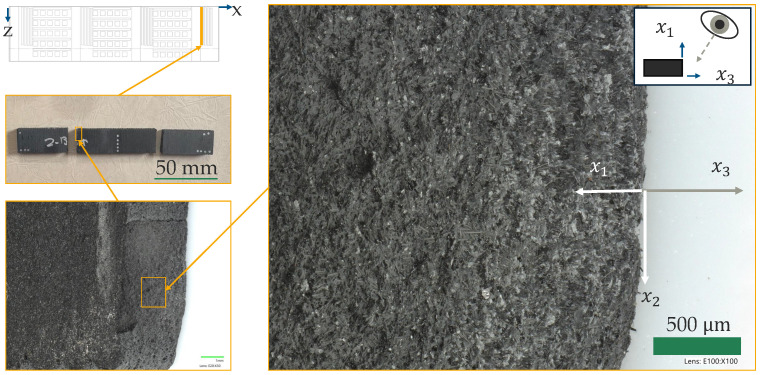
Fracture surface of Print 3—$x_{3}$ four-point bending Specimen 13.

A close-up view of an $x_{3}$ four-point bending specimen is shown in Figure 22. These images had to be taken at an angle to view the fracture surface. Compared to the fracture surface of the $x_{1}$ specimen, the fracture surface of the $x_{3}$ specimen is far smoother and appears to follow the layer boundary; however, it is difficult to determine the exact location of the layer interface in Figure 22. If the fracture occurred directly at the layer interface, the round perforations would be voids at the interface, which are also observed in Figure 11. The presence of interface voids points to another microstructure feature affecting the strength of the material. It follows that diffusion across the interface is not the only phenomenon relevant to the inter-layer strength, as there are stress concentration factors resulting from the inter-bead voids, and interface voids which agitate the state of stress at the interface and provide places for cracks to initiate.

### 3.5. Density Measurements

**Figure 23:**
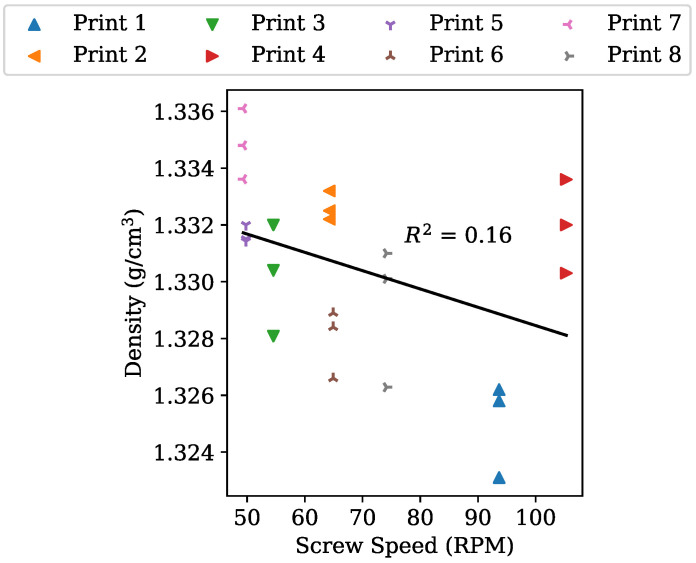
Density values based on gas pycnometer volume measurements.

The 24 density measurements from Prints 1 through 8 are shown in Figure 23. Average densities are given in Table 6. For reference, the density of the pellets as measured via a pycnometer (*N* = 3) ranged from 1.370 to 1.377 g/cm^3^. There was a very slight inverse correlation between ω and ρ, which agreed with the microstructure data.

## 4. Discussion

Although the results of this study offer insight into process–structure–property relations, it is useful to explore potential sources of variability, including microstructure, loading conditions, and experimental methods.

When measuring the microstructure, two major sources of uncertainty should be considered. The first is spatial uncertainty (e.g., variation in the local microstructure as a function of position in the print), and the second is the uncertainty associated with the analysis techniques used (e.g., accuracy in the process for measuring 3D fiber angle from a 2D image). Additionally, when measuring a 3D fiber orientation from a 2D image, there are two solutions to the orientation, meaning there could be asymmetric fiber orientation in the specimens that cannot be captured by 2D analysis.

A source of variability considered for the measured properties is microstructure. Specifically, the cut plan (cf. Figure 3) was consistent for all segments; however, the layer heights varied (cf. Table 1). Hence, locations of inter-bead voids varied within specimens of a given print and between specimens from different prints.

**Figure 24:**
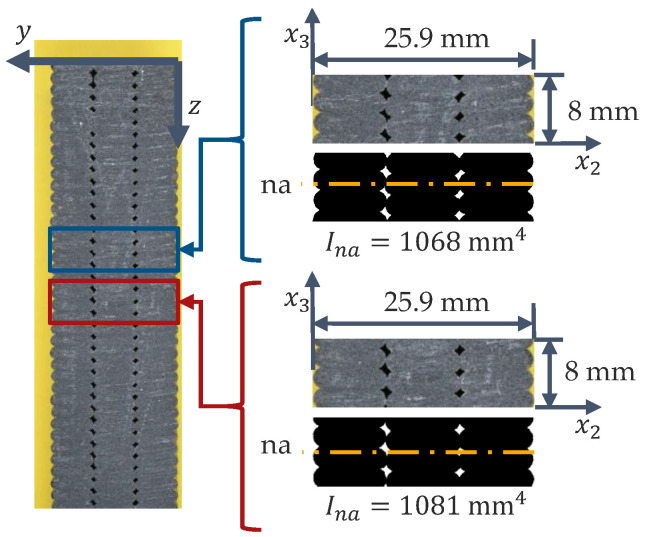
Image of two sections from Print 4 showing cut location, “specimen” thresholded image, neutral axis (na), and moment of inertia.

Among the prints, Print 4 had the greatest theoretical sensitivity in $x_{1}$ properties to the cut position based on the interaction between section moment of inertia and inter-bead void location. Figure 24 shows an annotated photo of Print 4, cross sections of two theoretical $x_{1}$ specimens, and thresholded images of two theoretical $x_{1}$ specimens with section properties.

An area moment of inertia for each theoretical $x_{1}$ bending specimen was calculated by thresholding each image to remove background and inter-bead void pixels. The remaining pixels representing material were summed and integrated to calculate the neutral axis (na) and moment of inertia about the neutral axis ($I_{na}$). The cross section was assumed to be symmetric.

The two values of $I_{na}$ are between the theoretical maximum of 1105 mm^4^ (considering a width of 25.9 mm and a thickness of 8 mm) for a specimen without inter-bead porosity and the theoretical minimum of 1040 mm^4^ (assuming a 25.9 mm by 8 mm specimen with the theoretical maximum inter-bead porosity for Print 4 of 5.87%, where voids are assumed to be uniformly distributed in the specimen). Moreover, the theoretical maximum volume fraction of inter-bead porosity $V_{f}^{max}$ for a given point in process space (assuming beads are rounded rectangles with varying corner radii) is given as

(3) $$V_{f}^{max}=\frac{d_{h}}{w}\left(1-\frac{\pi }{4}\right)$$

where $d_{h}$ is the layer height and *w* is the bead spacing. The results show that the variation resulting from cut location when considering the inter-bead porosity is likely minimal as the difference between the experimentally measured $I_{na}$ is 1.2%, whilst the difference between the theoretical bounds is 6%. Thus, the location of inter-bead voids and ridges relative to the cut locations fail to fully explain the observed intra-print variation in the modulus and strength in the $x_{1}$ direction.

As-measured property variability could also be caused by cut locations with regard to inhomogeneous intra-bead properties, where different regions of the bead exhibit different degrees of fiber orientation, thus, different moduli. This inhomogeneity is also non-deterministic as highlighted in Figure 11, where there is a region of lesser fiber alignment compared to other locations in the bead. Thus, inhomogeneous microstructure is a likely cause of some of the variability. This characteristic also extends along the bead, where the flow state at the nozzle is subject to variations resulting from the position of the screw in the extruder.

The complex state of stress near the loading noses in the four-point bending experiments represents another source of uncertainty. To understand the interactions between the complex state of stress around the loading noses and as-printed microstructure, further simulations with and without an explicit representation of microstructure are suggested.

The experimental methods also are possible sources of uncertainty. For example, the position of the specimen in the fixture, the position of the deflectometer, load cell precision, dimensioning the specimens, and possible slack in the fixtures are noted.

The experiments were explicitly designed to extract nominal microstructures and mechanical properties; thus, transients resulting from extruder starts and stops, path curves, and thermal effects from the bed would have negligible effects. Since the intended outcome is the measurement of nominal properties, the relative spatial invariance is a good reflection of representative conditions at the selected points in process space. Thus, the lack of any spatial correlations between cut location and strength are strong indications that much of the intra-print variation in the observed properties is not a result of predictable spatial variance, which is a sign that the observed intra-print variation is likely irreducible. Consequently, a nominal lower bound for a design COV for LSAM material $E_{11}$ is likely around 5%, which is invaluable for product design.

**Figure 25:**
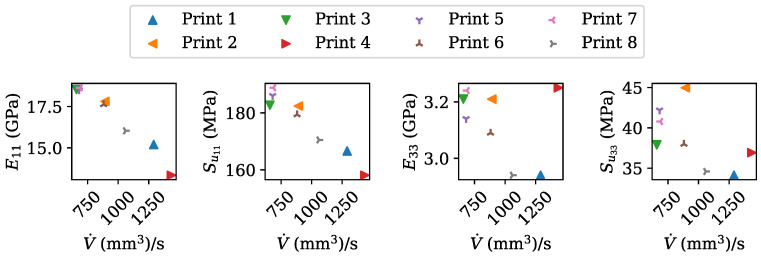
Mean $E_{11},S_{u_{11}},E_{33},S_{u_{33}}$ as a function of nominal volume flow rate $\dot{V}$.

The 18.6 GPa flexural modulus $E_{11}$ obtained for Print 7 is approximately 40% greater than the 13.3 GPa modulus obtained from Print 4. A plot showing mean properties and nominal volume flow rate $\dot{V}=d_{h}wf$ is shown in Figure 25. From a printing perspective, the actual volume flow rate is assumed to be primarily a function of the nominal screw speed ω.

The inverse relationship between nominal $\dot{V}$ and $E_{11}$ and $S_{u_{11}}$ is apparent in Figure 25. One possible explanation is the flow condition. Print 4 utilized a nominal $\dot{V}=d_{h}wf$ = (2.374 mm)(8.678 mm)(69.66 mm/s) = 1435 mm^3^/s, resulting in a mean nozzle velocity of $v_{nozzle}=\frac{\dot{V}}{0.25\pi d_{nozzle}^{2}}$ = 1435 mm^3^/s / (0.25π(6.0 mm)^2^) = 50.7 mm/s, where *f* is the feed rate, and $d_{nozzle}$ is the nozzle diameter, while Print 3 had a nominal $\dot{V}$ of 659.7 mm^3^/s and a corresponding $v_{nozzle}$ of 23.3 mm/s. The velocity at the orifice affects the flow conditions at the nozzle, where a greater velocity results in a greater Reynolds number, which generally decreases the influence of viscous effects (e.g., shear) relative to inertial effects [65]. Similar to wall friction inside a pipe, shearing flows inside an extruder nozzle tend to align fibers with the shearing direction [19]. Thus, a lower nozzle flow velocity is hypothesized to result in a higher degree of fiber alignment (cf. Figure 12), which in turn results in stronger and stiffer material (cf. Figure 16). These results are supported by the strong relationship between ω, $a_{11}$, and $E_{11}$. Another plausible cause of the hypothesized behavior is the observation that fibers align in the cross-flow direction in a diverging flow [19], like what happens when material leaves the nozzle and is shaped into the bead, and the stretching flows [14] that can occur in the same process.

Identifying sources of inter-print variations in $E_{33}$ according to the linear model were inconclusive, as the regression model lacked a sufficient $R^{2}$ value to be informative. Knowing that the more aligned the fibers are, the less isotropic a material is, allows us to infer that one plausible source of the inter-print variation is fiber alignment, and by decreasing the degree of fiber alignment, stiffness in the $x_{3}$ direction can be increased [19]. Moreover, there was a stronger correlation between $V_{f}^{I}$ and $E_{33}$. Results were inconclusive as to the driving mechanism behind inter-print variation in $S_{u_{33}}$, but a tenuous link between $V_{f}^{I}$ and $S_{u_{33}}$ was found, likely because of the effect of inter-bead porosity on the width of inter-layer interfaces. Thus, a possible method for increasing strength and stiffness in the $x_{3}$ direction is to over-extrude.

## 5. Conclusions

An experimental investigation was conducted to study and quantify the influence of process control parameters on microstructure and mechanical properties of eight cfPETg parts produced using large-scale additive manufacturing. The four processing parameters were bead spacing [6.112 mm to 8.678 mm], layer height [1.542 mm to 2.374 mm], feed rate [3685.9 mm/min to 4598.5 mm/min], and screw speed [50.53 RPM to 102.63 RPM]. Microstructure, with fiber orientation and porosity determined, was evaluated using microscopy. Flexural properties were measured via four-point bending, whereas density measurements were obtained via a pycnometer.

Overall, results indicated that an increase in screw speed resulted in a decrease in fiber alignment, which in turn resulted in a decrease in strength and stiffness along the bead. An increase in screw speed resulted in an increase in intra-bead porosity. Inter-layer flexural strength was associated with inter-layer interface width, with interface voids potentially driving inter-layer strength.

Future work would consist of (a) characterizing transient process effects, like the transients at the beginning and end of each raster, and the effects of curves, (b) creating a validation dataset for some physics-based or empirical model unbound to a specific material and printer combination, and (c) determining the primary mechanism driving fiber alignment for different material combinations. The creation and validation of such a model would enable more accurate predictions of as-printed properties for design, and a more informed material selection. Adding CTE to the measured properties would enable warpage control by modulating print parameters to control CTE via fiber orientation.

The work presented could be used to validate micro-mechanics models for LSAM and for validation of CFD-based models for process–structure–property relationships. Importantly, it was shown that samples were taken under nominal conditions, meaning the observed variability can serve as a basis for error bounds for PSP model validation. The quantity of data presented may serve as a training dataset for machine learning models for the prediction of strength, stiffness, and microstructure given process parameters. Finally, the most direct application of the work is the utilization of the measured quantities for cfPETg directly and using the findings to inform process parameters selection; overall, the following recommendations for improving as-printed properties are made: (1) print smaller beads slower when possible and (2) over-extrude to minimize the presence of inter-bead voids.

By characterizing LSAM process–structure–property relations, this and future studies facilitate the concurrent design of process space and part geometry. Concurrent design via optimization or optimization under uncertainty will allow engineers to design more efficient functional parts for aerospace, marine, and other applications. 

## Figures and Tables

**Table 1:** Print ID, processing parameters, die swell $d_{s}$, and aspect ratio $a_{r}$.

Print	$b_{s}$	$d_{h}$	*f*	ω	$d_{s}$	$a_{r}$
	(mm)	(mm)	(mm/s)	(RPM)	-	-
1	8.145	2.140	74.04	90.52	1.358	3.806
2	7.082	1.643	76.64	68.95	1.180	4.310
3	6.112	1.542	70.00	56.70	1.019	3.964
4	8.678	2.374	69.66	102.6	1.446	3.655
5	6.703	1.595	63.71	52.00	1.117	4.203
6	7.742	1.604	70.92	62.63	1.290	4.827
7	7.287	1.538	61.43	50.53	1.215	4.738
8	8.484	1.901	65.76	74.16	1.414	4.463

**Table 2:** Mean values and coefficients of variation of microstructure parameters based on samples from Prints 1 through 8.

		$V_{f}^{i}$		$a_{11}$		$a_{22}$		$a_{33}$	
Print	N	Mean	% COV	Mean	% COV	Mean	% COV	Mean	% COV
1	5	0.04	12.64	0.71	3.10	0.18	9.03	0.10	8.00
2	4	0.03	14.47	0.74	0.77	0.17	4.87	0.09	5.62
3	6	0.05	24.99	0.74	1.41	0.17	3.79	0.09	5.33
4	6	0.06	10.14	0.68	1.37	0.22	4.31	0.10	2.47
5	6	0.03	4.92	0.75	1.32	0.16	5.19	0.09	5.23
6	5	0.03	28.27	0.75	2.09	0.16	6.13	0.09	6.97
7	6	0.03	25.55	0.75	1.29	0.16	4.37	0.09	5.85
8	6	0.04	38.53	0.74	2.34	0.18	4.14	0.09	11.57
ALL	44	0.04	29.97	0.73	3.54	0.17	11.67	0.09	8.97

**Table 3:** Inter-bead porosity summary.

Print	$V_{f}^{I}\times 100$	(% COV)
1	3.37	18.47
2	0.11	98.06
3	0.02	163.49
4	2.41	8.99
5	0.24	11.51
6	1.07	20.01
7	0.08	173.64
8	3.24	16.61
ALL	1.44	101.57

**Table 4:** Print, flexural modulus, and strength via ASTM D6272 testing.

			$x_{1}$				$x_{3}$			
	N		$E_{11}$		$S_{u_{11}}$		$E_{33}$		$S_{u_{33}}$	
Print	$x_{1}$	$x_{3}$	Mean (GPa)	% COV	Mean (MPa)	% COV	Mean (GPa)	% COV	Mean (MPa)	% COV
1	12	11	15.20	2.74	166.6	5.10	2.94	6.04	34.12	8.35
2	13	11	17.82	4.46	182.4	3.17	3.21	3.99	44.97	9.43
3	11	10	18.54	6.16	182.7	4.21	3.21	3.65	37.90	6.90
4	15	12	13.34	5.26	158.1	3.90	3.25	4.50	36.92	3.80
5	11	10	18.51	2.23	186.0	3.01	3.14	3.25	42.19	7.35
6	13	10	17.63	1.88	179.4	2.83	3.09	3.56	37.99	6.64
7	9	10	18.64	4.23	188.8	4.62	3.24	3.80	40.78	8.08
8	12	6	16.03	1.59	170.5	2.27	2.94	4.40	34.58	8.27
All	96	80	16.91	11.96	176.6	7.04	3.12	5.75	38.61	11.92

**Table 5:** Mean values and coefficients of variation of strain at failure from ASTM D6272 testing.

	$x_{1}$		$x_{3}$	
Print	$\epsilon_{u_{11}}\times 10^{3}$	%COV	$\epsilon_{u_{33}}\times 10^{3}$	%COV
1	16.35	6.80	11.32	0.90
2	15.92	5.41	13.44	12.68
3	15.03	4.83	11.63	0.74
4	18.17	6.71	10.81	8.53
5	15.34	5.31	13.24	0.85
6	15.26	7.67	11.71	9.58
7	15.42	6.42	12.35	1.19
8	16.09	2.95	11.49	10.32
All	16.03	8.62	11.96	10.76

**Table 6:** Density measurements.

Print	Density (g/cm^3^)
1	1.325
2	1.333
3	1.330
4	1.332
5	1.332
6	1.328
7	1.335
8	1.329

## Data Availability

The original contributions presented in this study are included in the article. Further inquiries can be directed to the corresponding author.
